# Fecal carriage of extended-spectrum β-lactamases and AmpC-producing Escherichia coli in a Libyan community

**DOI:** 10.1186/1476-0711-13-22

**Published:** 2014-06-16

**Authors:** Salwa Fouad Ahmed, Mostafa Mohamed M Ali, Zienat Kamel Mohamed, Tarek A Moussa, John D Klena

**Affiliations:** 1 United States Naval Medícal Research Unit No.3, Cairo, Egypt; 2 Department of Microbiology and Immunology, Faculty of Medicine, University of Tripolí, Tripoli, Libya; 3 Department of Botany, Faculty of Science, Cairo University, Cairo, Egypt

**Keywords:** ESBLs, Libya, CTXM, AmpC, Fecal carriage, *E. coli*, MLST

## Abstract

**Background:**

Extended-spectrum β-lactamases (ESBLs), including the AmpC type, are important mechanisms of resistance among *Enterobacteriaeceae*. CTX-M type extended-spectrum β- lactamases, of which there are now over 90 variants, are distributed globally, yet appear to vary in regional distribution. AmpC β-lactamases hydrolyze third generation cephalosporins, but are resistant to inhibition by clavulanate or other β-lactamase inhibitors *in vitro*. Fecal carriage and rates of colonization by bacteria harboring these resistance mechanisms have been reported in patients with community-acquired infections and in healthy members of their households. Expression of these ESBLs compromises the efficacy of current antibacterial therapies, potentially increasing the seriousness of hospital- and community-acquired *Escherichia coli* (*E. coli*) infections.

To investigate the occurrence of ESBL-producing *E. coli* in human fecal flora isolated from two pediatric populations residing in the Libyan cities Zleiten and Abou El Khoms. Isolates were further studied to characterize genes encoding β-lactam resistance, and establish genetic relationships.

**Methods:**

Antibiotic resistance profiles of phenotypically characterized *E. coli* isolates recovered from the stools of 243 Libyan children during two surveillance periods in 2001 and 2007 were determined by the disk diffusion method. ESBL-screening was performed using the cephalosporin/clavulanate double synergy disc method, and the AmpC-phenotype was confirmed by the aminophenyl-boronic acid test. ESBL genes were molecularly characterized. Phylogenetic group and multilocus sequence typing (MLST) were determined for ESBL-producing isolates and PFGE was performed to compare banding profiles of some dominant strains.

**Results:**

ESBLs were identified in 13.4% (18/134) of *E. coli* isolates, and nine isolates (6.7%) demonstrated AmpC activity; all 18 isolates contained a CTX-M gene. Three CTX-M gene families (CTX-M-1, n = 9; CTX-M-15, n = 8 and CTX-M-3, n = 1) were distributed in diverse *E. coli* backgrounds (phylogenetic group D, 39%; B2, 28%; B1, 22% and A, 11%). MLST analysis revealed 14 sequence type (ST) with six new sequence types. The gene encoding the CMY-2 enzyme was detected in five AmpC-positive *E. coli*.

**Conclusions:**

These results identified heterogeneous clones of CTX-M-producing *E. coli* in the fecal isolates, indicating that the intestinal tract acts as a reservoir for ESBL-producing organisms, and a trafficker of antibiotic resistance genes.

## Background

The emergence of extended-spectrum-β-lactamase (ESBL)-producing *E. coli* in the community has been one of the most significant epidemiologic changes in infectious disease in recent years. The epidemiology of ESBL-associated infections is complex and combines the expansion of mobile genetic elements with specific clonal dissemination [1,2]. In the last decades, worldwide, *Enterobacteriaceae* producing ESBLs of the CTX-M type have shown a rapid and alarming dissemination and have been recognized as the most prevalent causative agents of hospital- and community-acquired infections [3-5]. The CTX-M- β lactamases, of which there are now over 150 different types detected in very distant regions such as Europe, the Near and Far East, South America, North America, and more recently in Africa are responsible for the recent huge increment in ESBLs throughout the world [3,6-10]. With the wide distribution of these enzymes worldwide, it seems that there are predominant enzymes in each region (3). However, there is limited data regarding the molecular epidemiology of ESBL-producing *Enterobacteriaceae* in the Middle East and North Africa (MENA) [11,12].

Several studies have demonstrated the high prevalence and diverse clones of CTX-M-producing *E.coli* from healthy humans [13,14] and have reported the rate of intestinal colonization by commensal *E. coli* isolates harboring ESBLs in healthy volunteers [15-18]. Despite the serious threat encountered by the transmission of these potentially harmful organisms in the community among healthy members of the same family unit, the rate of fecal carriage of ESBL-producing *Enterobacteriaceae* has mainly been investigated as part of nosocomial outbreak investigations [19] and the number of prospective longitudinal studies conducted in the hospital or in community settings remains rare [20]. In Libya, there is no a single report on the spread of bacterial resistance due to ESBL-producing isolates from either the hospital or community setting. We conducted the present study to investigate the occurrence of ESBL-positive *E. coli* isolates in human fecal flora and to characterize their encoding genes, and the clonal relationships.

## Methods

### Bacterial isolates

The research study was approved by U.S. Naval Medical Research Unit No. 3 in compliance with applicable Federal regulations governing the protection of human subjects. Informed consents were obtained from parents or legal guardians of minors. Stool samples were collected from 243 diarrheal libyan children aged from 3–12 years old at Al-Shafa Private Clinic and outpatient clinics of two Central Hospitals in Zliten and Alkhomes cities between April 2000 and March 2001 and the summer of 2007. A clinical history for each patient was obtained by an attending medical doctor and clinical symptoms, including fever, vomiting, and dehydration were recorded in a standard form. Within two hours of collection, stool specimens were directly streaked onto Mac-Conkey agar (Difco, Detroit, MI, USA) for isolation of *E. coli*. After overnight incubation at 37°C, 3 lactose-fermenting colonies were selected from each case and identified by the API 20E System (Biomerieux, SA, Marcy, l’Etoile, France).

### Antibiotic susceptibility and ESBL detection

Bacterial susceptibility to antibiotics was determined using the Kirby-Bauer agar disc diffusion method with bacterial isolates cultured on Mueller–Hinton agar. Susceptibility to ampicillin (10 μg), cephalothin (30 μg), chloramphenicol (30 μg), nalidixic acid (10 μg), streptomycin (10 μg), tetracycline (30 μg) trimethoprim-sulphamethoxazole (25 μg) ciprofloxacin (5 μg), ceftriaxone (30 μg), amoxicillin/clavulanate (30 μg) cefotaxime (30 μg), cefotaxime/clavulanate (30 μg/10 μg), ceftazidime (30 μg), ceftazidime/clavulanate (30/10 μg), ampicillin sulbactam (20 μg) and imipenem (10 μg) was determined; all antibiotics were purchased from Oxoid (Milan, Italy). Results were interpreted using published criteria from the Clinical Laboratory Standards Institute (CLSI) [21]. For reporting, intermediate and susceptible isolates are referred to as “non-resistant. Multi-drug resistance was defined as resistance to at least three families of antibiotics. ESBL activity was screened using the double-synergy test [22]. *E. coli* ATCC 25922 and *Pseudomonas aeruginosa* ATCC 27853 were used as control strains for susceptibility studies as recommended by CLSI 2011 guidelines for *Enterobacteriaceae*[21]. Phenotypic detection of AmpC production was performed using an amino-phenyl-boronic acid (APB)-based test as previously described [23].

### ESBL and AmpC characterization

Total bacterial DNA was purified using QIAmp DNA mini kits (Qiagen, Valencia, CA, USA) according to the manufacturer’s instructions. Genes encoding β-lactamase enzymes were amplified using previously described primers [24,25]. PCR products were purified using QIAquick PCR purification kits (Qiagen) following the manufacturer’s instructions. Sequencing reactions were carried out using the Perkin Elmer Big Dye kit v3.1 (Applied Biosystems, Foster City, CA, USA). Sequence data were collected using an ABI Prism 3100 automated sequencer, coupled to data collection software version 2.0, and sequencing analysis software 5.1.1 (Applied Biosystems). Nucleotide sequences obtained were assembled using BioEdit (version 7.0.5.3) and aligned with GenBank reference sequences using the Clustal X application within BioEdit.

### Molecular typing and phylogenetic groups of ESBL-positive isolates

The phylogenetic grouping of ESBL-positive *E. coli* isolates was determined using a previously described PCR-based method [26]. MLST was performed on all 18 CTX-M-producing ESBL isolates using the *E. coli* MLST scheme described at http://mlst.ucc.ie/mlst/dbs/E.coli, based on sequencing of internal regions of the housekeeping genes *adk, fumC, gyrB, icd, mdh, purA*, and *recA*[27]. CTX-M-1-producing *E. coli* isolates with a dominant MLST sequence type were further characterized using pulsed-field gel electrophoresis (PFGE) following a standardized protocol established by PulseNet USA, U.S. Centers for Disease Control and Prevention, for *E. coli* O157:H7 (PulseNet, USA) [28].

### Data analyses

Nucleotide sequences obtained from various isolates were aligned with GenBank reference sequences using the Clustal X application within BioEdit software (version 7.0.5.3), and compared with published sequences in the Lahey organization (http://www.lahey.org/studies). Data were statistically described in terms of frequencies (number of cases) and relative frequencies (percentages). Fisher’s exact test was used for comparison, a probability value (P value) less than 0.05 was considered statistically significant. All statistical calculations were done using Microsoft Excel 2007 (Microsoft Corporation, New York, USA), SPSS (Statistical Package for the Social Science; SPSS Inc., Chicago, IL, USA) or Quick Calcs Online calculators for scientists (Graph pad software Inc., San Diego, CA, USA).

## Results

### Detection of ESBL-producing isolates

One hundred and thirty-four confirmed *E. coli* isolates were recovered from the stools of 243 Libyan children during the two study periods (Table 1). Sixteen isolates from 2001 and five isolates from 2007 of the total 134 *E. coli* were from diarrheagenic cases (previously tested positive for virulence characteristics (29); the remaining 113 isolates were from non- diarrheagenic *E. coli* (negative for virulence factors [29]). Resistance to third generation cephalosporins (e.g., cefotaxime, ceftriaxone and ceftazidime) was observed in 20% of isolates, more often observed in the first surveillance period (2000–2001). Of the 14 antimicrobial agents tested, *E. coli* isolated from Libyan children in 2001 were significantly more resistant to cephalothin, cefotaxime, ceftriaxone, ceftazidime, chloramphenicol, nalidixic acid and Ciprofloxacin than total *E. coli* from Libyan children in 2007 (Table 1). Overall, the percentage of ESBL positive isolates was 13.4% (18/134) with no significant occurrence (P = 0.2) in 2001 (14/85) as compared to 2007 (4/49) (Table 2). The AmpC phenotype was detected in 6.7% (9/134) of *E. coli* and was mainly associated with *E. coli* isolates from 2001 isolates (Table 2). In addition to the 27.8% (5/18) resistance to both NA and CIP, ESBL-positive isolates displayed a significant higher level of multi-drug resistance to 12 of 14 antimicrobial agents tested as compared to ESBL-negative isolates (Table 3).

**Table 1:** **Antibiotic resistance of *Esherichia coli *** **strains isolated from Libyan children during 2001 & 2007**

**Antibiotic**	**2001**	**2007**	P-values*	**Total**
	**(N = 85)**	**(N = 49)**		**(N = 134)**
Ampicillin	69 (81.2)**	36 (73.5)	0.3	105 (78.4)
Ampicillin/Sulbactam	50 (58.8)	26 (53.1)	0.5	76 (56.7)
Amoxicillin/Clavulanic	54 (63.5)	32 (65.3)	0.8	86(64.2)
Imipenem	0	0	N/A	0 (0.0)
Cephalothin	67 (78.8)	30 (61.2)	0.03	97 (72.4)
Cefotaxime	23 (27.1)	4 (8.2)	0.009	27 (20.2)
Ceftriaxone	22 (25.9)	4 (8.2)	0.01	26 (19.4)
Ceftazidime	23 (27.1)	4 (8.2)	0.009	27 (20.2)
Chloramphenicol	39 (45.9)	8 (16.3)	0.0005	47(35.1)
Nalidixic acid	4 (4.7)	9 (18.4)	0.01	13 (9.7)
Ciprofloxacin	2 (2.4)	5 (62.5)	<0.0001	7 (5.2)
Streptomycin	57 (67.1)	28 (57.1)	0.2	85 (63.4)
Trimethoprim/sulfamethoxazole	54 (63.5)	29 (59.2)	0.6	83 (61.9)
Tetracycline	48 (56.5)	22 (44.9)	0.2	70 (52.2)

* Fisher’s exact test.

**Data presented as N (%) of resistance cases, where N: Number of cases displaying resistance.

N/A: Not applicable.

**Table 2:** **Extended-spectrum beta-lactamase and AmpC phenotypes of *Esherichia coli *** **strains in human fecal samples from Libyan children**

**Beta-lactamase phenotype**	**2001**	**2007**	P-Values*	**Total**
	**(N = 85)**	**(N = 49)**		**(N = 134)**
ESBL	14(16.5)**	4(8.2)	0.3	18(13.4)
AmpC	9(10.6)	0(0)	0.07	9(6.7)

* Fisher’s exact test.

**Data presented as N (%) of resistance cases, where N: Number of cases displaying resistance.

**Table 3:** **Antibiotic resistance of *Esherichia coli *** **according to extended-spectrum beta-lactamases phenotype in fecal samples from Libyan children**

	**ESBL + ve**	**ESBL-ve**	P-Value*	**Total**
	**(N = 18)**	**(N = ll6)**		**(N = l34)**
Ampicillin	18 (100)**	87 (75)	0.02	105 (78.4)
Ampicillin/Sulbactam	17 (94.4)	59 (50.9)	0.0005	76 (56.7)
Amoxicillin/Clavulanic	17 (94.4)	69 (59.5)	0.3	86 (64.2)
Imipenem	0 (0.0)	0 (0.0)	N/A	0 (0.0)
Cephalothin	18 (100)	79 (68.1)	0.003/	97 (72.4)
Cefotaxime	18 (100)	9 (7.8)	<0.0001	.27 (20.2)
Ceftriaxone	18 (100)	8 (6.9)	<0.0001	26 (19.4)
Ceftazidime	18 (100)	9 (7.8)	<0.0001	27 (20.2)
Chloramphenicol	17 (94.4)	30 (25.9)	<0.0001	47 (35.1)
Nalidixic acid	5 (27.8)	8 (6.9)	0.03	13 (9.7)
Ciprofloxacin	5 (27.8)	2 (1.7)	0.0009	7 (5.2)
Streptomycin	16 (88.9)	69 (59.5)	0.02	85 (63.4)
Trimethoprim/sulfamethoxazole	14 (77.8)	69 (59.5)	0.2	.83 (61.9)
Tetracycline	16 (88.9)	54 (46.6)	0.001	70 (52.2)

*Fisher’s exact test.

**Data presented as N (%), where N: Number of cases displaying resistance.

N/A: Not applicable.

### Characterization of blaESBL and blaAMPC in ESBL-producing isolates

PCR amplification and DNA sequence analyses of the 18 ESBL-producers showed that all possessed a CTX-M-type ESBL and revealed three families of *bla*CTX-M; *bla*CTX-M-1 (n = 9), blaCTX-M-15 (n = 8), and *bla*CTX-M-3 (n = 1). All of the CTX-M positive isolates were also positive for *bla*TEM, while 16.7% of isolates carried either *bla*SHV-2 or *bla*OXA-1 (Table 4). None of the CTX-M-1 family members demonstrated resistance to ciprofloxacin; however, a relationship with fluoroquinolone resistance was noted with ESBL-producing isolates harboring the *bla*CTX-M-15. The gene encoding the CMY-2 enzyme was detected in five AmpC-positive *E. coli*.

**Table 4:** **Characteristics of *Escherichia coli bla***_**CTXM **_**families- producing isolates in human fecal samples of Libyan children**

**Specimen #**	**Date of isolation**	***bla ***_***CTXM***_	***bla ***_***TEM***_	***bla ***_***OXA***_	***bla ***_***SHV***_	**Phylogenetic group**	ST*	PFGE Pattern**	**Antibiotic resistance profile**
L1	2001	1	+	-	-	D	New	A	Amp, SAM, AMC, CF, CTX, CRO, CAZ, C, S, SXT, TE
L2			+	-	-				Amp, SAM, AMC, CF, CTX, CRO, CAZ, C, S, SXT, TE
L3			+	-	-				Amp, SAM, AMC, CF, CTX, CRO, CAZ, C, S, SXT, TE
L4			+	-	-			ND§	Amp, SAM, AMC, CF, CTX, CRO, CAZ, C, S, SXT, TE
L5			+	-	-				Amp, SAM, AMC, CF, CTX, CRO, CAZ, C, S, TE
L6			+	-	-	B2	12	C	Amp, SAM, AMC, CF, CTX, CRO, CAZ, C, S, SXT, TE
L7			+	-	2				Amp, SAM, AMC, CF, CTX, CRO, CAZ, C, S, SXT, TE
L8			-	-	-			B	Amp, SAM, AMC, CF, CTX, CRO, CAZ, C, SXT
L9			+	-	2			C1	Amp, SAM, AMC, CF, CTX, CRO, CAZ, C, S,TE
L10		3	+	-	-	A	23	ND§	Amp, CF, CTX, CRO, CAZ, C, S,TE
L11		15	+	-	2	B2	127		Amp, SAM, AMC, CF, CTX, CRO, CAZ, C, S, SXT, TE
L12			+	1	-	B1	359	D	Amp, SAM, AMC, CF, CTX, CRO, CAZ, C, NA,CIP,S, SXT, TE
L13			+	1	-			ND§	Amp, SAM, AMC, CF, CTX, CRO, CAZ, C, NA,CIP,S, SXT, TE
L14			+	-	-	D	New		Amp, SAM, AMC, CF, CTX, CRO, CAZ, C, S, SXT, TE
L15	2007		+	-	-	B1	155		Amp, SAM, AMC, CF, CTX, CRO, CAZ, C, NA,CIP,S, SXT, TE
L16			+	-	-	D	38		Amp, SAM, AMC, CF, CTX, CRO, CAZ, C, S, SXT, TE
L17			+	-	-	B1	46		Amp, SAM, AMC, CF, CTX, CRO, CAZ, C, NA,CIP,S, SXT, TE
*L18*			+	1	-	A	410		Amp, SAM, AMC, CF, CTX, CRO, CAZ, NA,CIP

*ST: Sequence type as analyzed by multi-locus sequence typing (MLST).

**PFGE : Pulse-Field gel electrophoresis.

§ND: Not done.

Amp, ampicillin; SAM, sulbactam-ampicillin; AMC, amikacin; CF, cephalothin; CTX, cefotaxime; CRO, ceftraxione; CAZ, ceftazidime; C, chloramphenicol; NA, nalidixic acid; CIP, ciprofloxacin; S, streptomycin; SXT, sulfamethoxazole/trimethoprim; TE, tetracycline.

### Molecular typing of ESBL-positive isolates

The genetic background of the 18 ESBL positive isolates revealed their distribution into four phylogenetic groups (Table 4): 39% (7/18) were group D, 28% (5/18) were group B2, and 22% (4/18) were group B1 while two isolates (11%) belonged to group A. Five of the nine *bla*CTX-M-1 isolates belonged to phylogroup D and the remaining four belonged to phylogroup B2. Because CTX-M-1 predominated during the first surveillance period, we were eager to study their genetic backgrounds. PFGE analysis on *bla*CTX-M-1 isolates identified three major band patterns (see Additional file 1): pattern 1 consisted of three isolates with indistinguishable macrorestiction profile (mrp), pattern 2 consisted of one isolate with an unique mrp, and pattern 3 included three isolates:2 isolates with indistinguishable mrp, and one isolate had a single band difference from pattern 3 and was named 3a. The unique CTX-M-15 producing isolates demonstrated a distinct pattern 4 from that displayed by CTX-M-1. MLST analyses revealed that the blaCTX-M-1 isolates with pattern 1 belonged to a new ST group, while those with profile 2, 3 and 3a belonged to ST12. MLST revealed an additional eight ST groups in which the remaining nine ESBL-producing isolates [CTX-M-15, (ST127, ST359 (n = 2), ST NEW, ST155, ST38, ST 46, ST410); CTX-M-3, (ST23)] were grouped (Table 4).

## Discussion

In this study we investigated the occurrence of ESBLs in human fecal flora from Libyan children living in two cities during two time periods. Overall, the percentage of fecal ESBL-producing *E. coli* recovered is comparable to those reported from other parts of the world including Saudi Arabia [30] and Tunisia [14], but lower than those found in Egypt [18], Thailand [17], China [13], and Japan [31]. In our study, the majority of ESBL-producing *E. coli* was isolated from patients with community-acquired infections and not from hospitalized patients with diarrhea which suggests the ability of ESBL-producing isolates to spread in the community beyond the hospital environment and potentially exclude their limitation to nosocomial infections, thus helping to exacerbate public health concerns [5]. Leflont et al. [32] have recently reported the increased rate of ESBL fecal carriage in healthy subject populations living in Paris from a 0.6% to 6% over a five year period and indicated that acquisition from a common source related to food and person-to-person transmission may contribute to the spread of ESBL-producing *E. coli* within families.

The presence of these organisms in the bowel in high rates was suggested to be a risk factor for infections with these bacteria and their dissemination in fecal flora [33]. In developing countries most patients received antimicrobial treatment without prescription, such common practices in nearly all developing countries exert a selective pressure on *E. coli* organisms, known to be a common cause of community-acquired infections, [34] to develop such resistance that leads to a potential sustainable source for nosocomial infections. In our study, *bla*CTX-M gene was detected in all 18 ESBL-producing *E. coli* isolates and *bla*CTX-M-1 was the most commonly identified resistance mechanism in samples from the earliest surveillance point (April 2000 to March 2001). Of particular interest in this region, the existence of three types of CTX-M families identified during the first year of study with a preponderance of CTX-M-1 in previous year and a persistence of CTX-M-15 later.

This finding supports work by others indicating that a specific CTX-M enzyme can dominate in a geographical region [3]. A possible explanation could be due to the dynamic nature of organisms harboring these enzymes; over time the dominant mechanism will change, as the dominant clonal group changes. While CTX-M-15 is the most common CTX-M type in the Middle East region and North Africa, other investigators have reported that CTX-M-15 was not more prevalent than CTX-M-1 among the fecal isolates [16,35]. The difference in distribution of CTX-M types was also attributed by some studies to the various sources of the CTX-M-producing *E. coli* isolates carried in the gut of patients [15]. Some previous reports suggested a possible transmission of ESBL-producing *E. coli* through the food chain [36]; interestingly, it was shown, particularly in France, that CTX-M-1 is the predominant CTX-M among food-producing animals [37].

In general, CTX-M-producing *E. coli* have predominantly been identified from the community as a cause of urinary tract infections. However, the high rate of their fecal shedding in the outpatient setting in Libya is an emerging epidemiological problem that requires application of updated control measures like routine rectal screening of patients admitted from the community, even though routine fecal examination sounds impractical and is not required even if the patients were admitted to an intensive care unit in most hospitals and institutions around the world [38].

It is worth-mentioning that CTX-M β-lactamase genes are widely known to be carried on a plasmid linked to mobile genetic elements that are utilized as vehicles for resistance genes horizontal movement, in addition to carry on resistance to other drug like aminoglycoside and fluoroquinolone [15,39,40]. CTX-M isolates from the current study demonstrated resistance to non-β –lactam classes including the aminoglycoside and fluoroquinolone families.

The coexistence of AmpC phenotype in an ESBL isolate is a serious phenomenon that can mask the accurate detection of the ESBL phenotype [41]; this might have a critical effect on the success of clinical treatment. In our study, nine CTX-M positive isolates exhibited AmpC phenotype and CMY-2 was the main gene detected. Other AmpC genes previously reported (23) were not tested for the remaining negative samples. A recent study from Egypt has reported the occurrence of both resistance mechanisms for ESBL *Enterobacteriaceae* from outpatients and hospitalized cases, recommending the proper use of modified double-disc synergy test (MDDST), in addition to confirmatory PCR testing in order to avoid the unsuccessful identification of such isolates when they coexist [23].

Multiple studies have associated the dissemination of ESBL-CTX-M-15-producing isolates with the spread of the epidemic ST131 *E. coli* strain belonging to phylogenic group B2 [12,33]. Interestingly, our phylogenetic group analysis demonstrated that most of CTX-M-15 positive isolates did not fall under B2 group, and according to the MLST typing system, epidemic ST131 clone, most globally ST associated with CTX-M-15, was not identified, indicating that this clone is less represented in fecal ESBL-producing *E. coli*, which is in agreement with other recent studies from Tunisia [15] and Spain [36]. In contrast, the present study identified seven ST clones associated with the dissemination of CTX-M-15 that are not previously implicated in isolates producing-ESBL, in addition to two emerged ST reported earlier in Spain (ST155) and Tunisia (ST23) from fecal *E. coli* with carbapenamases and ESBL activites, respectively. This particular characteristic of Libyan CTX-M-15 isolates indicates that their dissemination occurred by heterogeneous clones with a different genetic background.

In summary, this is the first detailed description of fecal CTX-M-producing *E. coli* in Libya. The dynamic nature and high clonal diversity of fecal CTX-M-producing isolates amplify the importance of the intestinal tract to act as a reservoir for ESBL-producing organisms, and a trafficker of antibiotic resistance genes. The increased prevalence of these organisms in human fecal flora and their establishment in the community represent an opportunity for these clones to become persistent. Because of the significant public health implications, the spread of organisms producing CTX-M-producing isolates merits close monitoring with enhanced surveillance efforts [31], with the introduction of molecular diagnostic procedures in a clinical or reference laboratory to track their spread in the community and hospital settings.

### Limitation

Although the number of isolates studied is too low to obtain a clear conclusion, coupled with the lack of identifiable risk factors, we cannot underscore the potential risk associated with the dissemination of these organisms in Libyan hospitals and the community at large. Therefore, a wider hospital and population-based surveillance study should be implemented over a distinct period of time to be able to truly reflect the molecular epidemiology of CTX-M-producing *E. coli* strains on a country-wide scale. In addition, the true reservoir of CTX-M-producers *E. coli* in normal healthy individuals should be investigated.

## Competing interests

The authors declare that they have no competing interests.

## Authors’ contributions

SFA and JDK designed and supervised molecular genetic studies, and wrote the manuscript. MMA carried out the molecular genetic studies and participated in data analysis. ZKM and TAM reviewed the manuscript and data content. All authors read and approved the final manuscript.

## Supplementary Material

Additional file 1 **Mrp: Macro-restriction pattern as determined by PFGE.** ST: Sequence type as analyzed by MLST. *E.coli* gp: *E.coli* Phylogenetic group. Lane1-4: CTX-M-producing *E.coli* isolates (L1, L8, L5 & L2); lane5-8 (L6, L7, L9 & L12); lane 9: *S.branderup.* Click here for file
