# Supplementary material for: Fecal carriage of extended-spectrum β-lactamases and AmpC-producing Escherichia coli in a Libyan community
Source: Ann Clin Microbiol Antimicrob. 2014 Jun 16;13:22. doi: 10.1186/1476-0711-13-22 (PMC4107601; doi:10.1186/1476-0711-13-22)

**Additional File 1.**

**Figure S1**. PFGE of selected bla CTXM-1 *E.coli*-producing isolates.

mrp 1 2 1 1 3 3 3a 4

ST New ST12 New New ST12 ST12 ST12 ST359

*E.coli* gp D B2 A A B2 B2 B2 B1

Lane No. 1 2 3 4 5 6 7 8 9


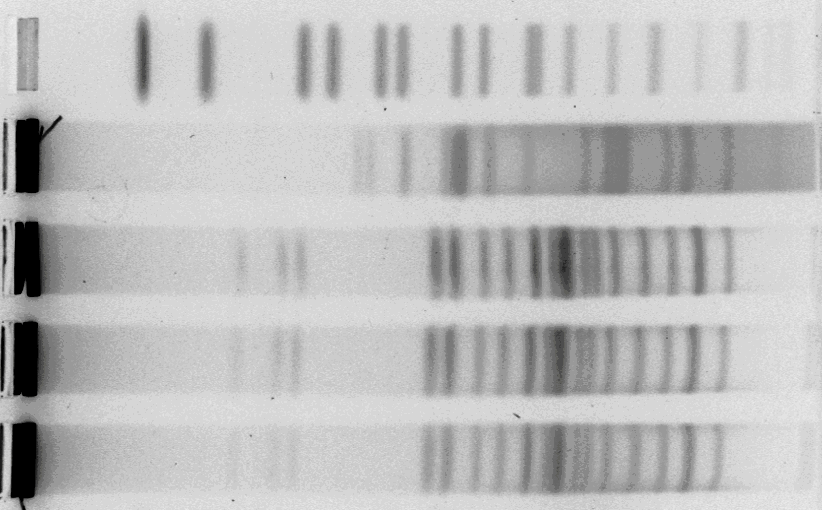

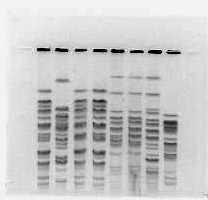

Supplement: Additional file 1 — Mrp: Macro-restriction pattern as determined by PFGE. ST: Sequence type as analyzed by MLST. E.coli gp: E.coli Phylogenetic group. Lane1-4: CTX-M-producing E.coli isolates (L1, L8, L5 & L2); lane5-8 (L6, L7, L9 & L12); lane 9: S.branderup. [file 1476-0711-13-22-S1.docx]
